# Decreased FGF19 and FGF21: possible underlying common pathogenic mechanism of metabolic and cognitive dysregulation in depression

**DOI:** 10.3389/fnins.2023.1165443

**Published:** 2023-05-17

**Authors:** Mimi Tang, Shuqiao Cheng, Lu Wang, Hui Tang, Ting Liu, Tingyu Zhao, Ruili Dang

**Affiliations:** ^1^Department of Pharmacy, Xiangya Hospital, Central South University, Changsha, China; ^2^Institute for Rational and Safe Medication Practices, National Clinical Research Center for Geriatric Disorders, Xiangya Hospital, Central South University, Changsha, China; ^3^Mental Health Institute of the Second Xiangya Hospital, Central South University, Changsha, China; ^4^Translational Pharmaceutical Laboratory, Jining First People’s Hospital, Jining Medical University, Jining, China

**Keywords:** FGF19, FGF21, metabolic dysregulation, cognitive dysregulation, depression

## Abstract

**Background:**

Accumulating studies suggested that major depressive disorder (MDD) was closely related to metabolic syndrome (MetS). Important endogenous regulators fibroblast growth factors (FGFs) 19 and 21 were also reported to participate in psychiatric disorders. This study aimed to investigate the role of FGF19 and FGF21 in MDD and to explore the possible pathogenic mechanism of metabolic and cognitive dysregulation in depression.

**Methods:**

A total of 59 MDD patients and 55 healthy control participants were recruited. The serum levels of FGF19 and FGF21 and lipid profiles were measured by means of enzymatic methods. Cognitive function was measured by repeatable battery for the assessment of neuropsychological status (RBANS) scores. The gene expression of PGC-1α and FNDC5 was determined by quantitative polymerase chain reaction (PCR).

**Results:**

We found that plasma FGF19 and FGF21 levels were significantly decreased in patients with MDD. Meanwhile, triglyceride (TG) was significantly elevated and PGC-1α was significantly downregulated in MDD patients. Correlation analyses showed negative associations between TG and FGF19 levels. As for cognitive performance, both FGF19 and FGF21 levels were positively correlated with immediate memory. However, FGF19 levels were negatively correlated with language, and FGF21 levels were also negatively correlated with attention and delayed memory. Additionally, negative associations were found between FGF19 levels and PGC-1α. FGF21 levels were positively associated with PGC-1α and negatively associated with FNDC5.

**Conclusion:**

This study elucidated the role of FGF19 and FGF21 in MDD. MDD patients were confirmed to have metabolic and cognitive dysregulation, and this abnormality was linked to the decreased concentrations of FGF19 and FGF21 through the PGC-1α/FNDC5 pathway. Our results showed that the alterations of FGF19 and FGF21 levels may be a common pathogenic mechanism of metabolic and cognitive disturbances in patients with MDD.

## Introduction

Major depressive disorder (MDD) is a commonly occurring mental disorder and is estimated to be the leading cause of disability worldwide (Malhi and Mann, 2018). Another big health threat is metabolic syndrome (MetS), which is defined as a clustering of obesity, insulin resistance (IR), hypertension, and dyslipidemia (Penninx, 2016). Epidemiological studies have consistently reported a high rate of co-morbidity between these two most common and debilitating disorders, with an estimated prevalence of MetS as high as 48% among MDD patients (An et al., 2012). In cohort studies, MetS and its components have been associated with more severe clinical depressive symptoms and a higher risk of MDD (Sagud et al., 2009; Hummel et al., 2011; Scharnholz et al., 2014). Meanwhile, several meta-analyses of cohort studies suggested that individuals with MDD had a significantly increased risk for MetS and its components compared to matched general population control (Vancampfort et al., 2014, 2015). The association between MetS and MDD is complex and remains incompletely understood, and the bidirectional correlations between them suggest a possible pathophysiological overlap.

Fibroblast growth factors (FGFs) 19 and 21 belong to a subfamily of FGFs that function as hormones, regulating a plethora of biological functions, including energy homeostasis and brain development (Beenken and Mohammadi, 2009). Human FGF19 is a gut-derived circulating hormone, while FGF21 is mainly produced by the liver during metabolic stress. FGF21 has been shown to play a key role in the control of many aspects of energy homeostasis in both preclinical and clinical studies, and it represents an interesting candidate for the treatment of obesity and type 2 diabetes (Dolegowska et al., 2019). In mouse models of obesity and type 2 diabetes, treatment with FGF21 improves glucose homeostasis and promotes weight loss (Jimenez et al., 2018). Pharmacologically, FGF19 shows similar antiobesity and antidiabetic actions in rodent models as FGF21, with these metabolic effects being partly mediated by the brain (Gadaleta and Moschetta, 2019).

During the past decade, accumulating evidence suggests the involvement of FGF 19 and 21 in psychiatric disorders. A negative correlation between the cerebrospinal fluid (CSF) FGF21 level and depressive symptoms has been reported (Liu et al., 2017a). Furthermore, FGF21 has been shown to reduce lipopolysaccharide-induced depressive-like behaviors in rodent models, while FGF21-deficient mice showed augmented depressive-like behaviors (Usui et al., 2021). Another study reported that serum levels of FGF21 in depressed bipolar disorder are affected by mood-stabilizing agents (Leng et al., 2015). FGF19 has been shown to involve in cell proliferation and cell survival during embryonic brain development (Somm and Jornayvaz, 2018). Recently, significant correlations were found between human CSF FGF19 levels and Beck Depression Inventory scores (Liu et al., 2017b). These studies indicated that FGF19 and FGF21 are involved in the development of depression.

Previous studies reported the important metabolic mediators, peroxisome proliferator activated receptor gamma (PPAR-γ) coactivator 1-alpha (PGC-1α) and fibronectin type III domain containing protein 5 (FNDC5), are activated by endurance exercise, inducing the brain-derived neurotrophic factor (BDNF) expression in the brain. FNDC5 was identified as a PGC-1α-dependent myokine, which promotes the browning of beige fat cells in white adipose tissue and mediates some of the major metabolic benefits of exercise. Importantly, peripheral delivery of FNDC5 via adenoviral vectors induced the expression of BDNF and other neuroprotective genes in the hippocampus of mice (Wrann et al., 2013; Zhan et al., 2018). These data indicate that the PGC-1α/FNDC5 pathway may represent an interesting connection between energy homeostasis and brain function. FGF21 was shown previously to induce hepatic expression of PGC-1α, suggested as a mechanism for its metabolic actions (Fisher et al., 2012). A previous study showed that exercise or cognitive training had an impact on both BDNF and irisin (the secreted form of FNDC5) serum levels that positively correlated with global cognition scores and memory (Küster et al., 2017). This has a significant implication to establish novel blood-based biomarkers of cognition and brain function.

In this study, we measured the serum levels of FGF19 and FGF21, assessed the PGC-1α/FNDC5 pathway in patients with MDD, and compared this to results from healthy controls. These findings were used to investigate the relationship between these factors and depression with co-morbid metabolic diseases and to explore the underlying developmental mechanisms.

## Materials and methods

### Participants

A case–control clinical study was conducted at the Mental Health Institute of the Second Xiangya Hospital at the Central South University, China. The study protocols were approved by the hospital’s clinical research ethics committee, and each of the participants signed informed consent.

A total of 59 patients who met the Diagnostic and Statistical Manual of Mental Disorder-Fourth Edition (DSM-IV) criteria for MDD and 55 healthy control participants were recruited from September 2016 to September 2019. All patients should meet the following criteria as described in our previously published article (Wang et al., 2020): patients (1) who aged between 18 and 50 years, (2) who were first diagnosed, drug-naive, and physically healthy, and (3) whose Hamilton Depression Rating Scale (HAMD) score is −24 ≥ 21. Exclusion criteria included those as follows: (1) patients with any other psychiatric disorders other than MDD, (2) patients with any physical diseases (i.e., organic brain diseases, hypertension, diabetes mellitus, tumor, and thyroid disease), (3) patients with a history of familial psychiatric disorders, (4) pregnant or lactating women, (5) patients with an apparent suicide attempt or suicidal behavior, and (6) patients who need to take benzodiazepine every day, and who currently need to be treated by electroconvulsive therapy or have received electroconvulsive therapy in the past 6 months.

Healthy controls (HCs) recruited for this study who had no history of MDD or other mental disorders. A complete medical history and physical examination were obtained from all subjects, and any subjects with serious medical abnormalities were excluded. A clinician ruled out any mental illnesses in HCs by using Symptom Check List 90 (SCL90) and direct psychiatric interviews. HCs were group matched with study subjects for age, sex, and education.

### Clinical assessment and cognitive measures

Two experienced psychiatrists, blinded to the participant’s clinical status, assessed the participant’s psychopathology using a relevant scale. To ensure consistency and reliability of ratings, these two psychiatrists attended a training session for standardizing their use of the scales before the study. Mental status was assessed by psychiatrists using the HAMD and Hamilton Anxiety Rating Scale (HAMA). Beck Depression Inventory-II (BDI-II) and Self-rating Anxiety Scale (SAS) were used for patient self-assessment.

The repeatable battery for the assessment of neuropsychological status (RBANS) was administered to measure cognitive function. The RBANS is composed of 12 subtests that are used to calculate five age-adjusted index scores and a total score. Test indices are immediate memory (list learning and story memory), visuospatial/constructional (figure copy and line orientation tasks), language (picture naming and semantic fluency tasks), attention (digit span and coding task), and delayed memory (list recall, story recall, figure recall, and list recognition tasks). The RBANS test was performed within the same time frame for patients and controls.

### Blood sample collection and serum parameters measurement

The blood samples were centrifuged at 3000 rpm for 20 min at 4°C to separate the serum after being kept for 1 h at room temperature. The serum samples were aliquoted and stored at −80°C. The serum FGF19 and FGF21 were measured using the human FGF19 and FGF21 ELISA Kit, respectively (Antibody and Immunoassay Services, Hong Kong; Catalog Number: 31200) according to the manufacturer’s instructions. Serum levels of triglyceride (TG), total cholesterol (TCH), high-density lipoprotein-cholesterol (HDL-c), low-density lipoprotein-cholesterol, and (LDL-c) were measured by means of enzymatic methods, using assay kits (Sekisui Medic; Abbott Laboratories or Beijing Leadman Biochemistry Co., Ltd).

### Real-time PCR analysis

Total RNA was extracted from the blood using Trizol reagent (Invitrogen, United States) following the manufacturer’s instructions for the detection of the gene expression of PGC-1α and FNDC5. RNA concentration was determined for quantity and integrity using spectrophotometry (Jingke, China). cDNA was produced using the Revert Aid First Strand cDNA Synthesis Kit (Takara Bio, Japan). Quantitative PCR was performed on the Bio-Rad Cx96 Detection System (Bio-Rad, United States) using the SYBR green PCR kit (Takara Bio, Japan) and gene-specific primers. An amount of 5 mg cDNA sample was used with 40 cycles of amplification. Each cDNA was tested in triplicate. Relative quantitation for the PCR product was normalized to β-actin as an internal standard. The sequences of gene-specific primers are listed in Table 1.

**Table 1:** Sequences of gene-specific primers.

Gene	Primers-F	Primers-R
PGC-1α	TGGTGGACACGAGGAAAG	CTGCCAATCAGAGGAGACAT
FNDC5	ATCTCATCCAGGGTTCCA	CCTCATCAAGCACCATTT
GAPDH	TCAAGAAGGTGGTGAAGCAG	CAAAGGTGGAGGAGTGGGT

### Statistical analysis

Continuous variables were examined for normality by the Kolmogorov–Smirnov test and the coefficients of kurtosis and skewness. Data following normal distribution were expressed as the mean ± SD, and categorical variables were expressed as numbers. Data were analyzed using SPSS 19 (IBM Corporation, Armonk, New York, NY, United States), and figures were prepared using GraphPad Prism 8 (GraphPad Software, Inc., La Jolla, CA, United States). Clinical data were analyzed by the *t*-test or chi-square test, and the relationships between serum FGF19, FGF21, and metabolic and cognitive parameters were assessed using the Pearson correlation coefficient. The difference was considered to be statistically significant at a value of *p* <0.05.

## Results

### Characteristics of participants

A total of 59 first-diagnosed, drug-naïve patients with MDD and 55 healthy control participants were recruited. The demographic and clinical data are shown in Table 2. There were no significant differences between MDD patients and controls in sex distribution, age, education, and BMI, whereas depressed patients presented significantly higher BDI-II and SAS scores than the controls (*p* < 0.001).

**Table 2:** Clinical data of participants.

	MDD (*n* = 59)	HCs (*n* = 55)	t/*X*^2^	*p*-value
Males/females	28/31	24/31	0.904	0.342
Age (years)	27.55 ± 8.09	29.11 ± 8.84	0.97	0.335
Education (years)	13.79 ± 3.22	14.11 ± 2.90	0.53	0.600
BMI (Kg/m^2^)	21.08 ± 3.84	22.01 ± 2.70	1.45	0.151
BDI-II scores	27.15 ± 9.30	7.85 ± 5.73	−11.17	**<0.001**
SAS scores	47.74 ± 8.79	31.93 ± 6.11	−7.47	**<0.001**
HAMD scores	26.96 ± 7.49	–	–	–
HAMA scores	20.36 ± 7.63	–	–	–

Data were expressed as mean ± sd. MDD, major depressive disorder; HCs, healthy controls; BDI-II, Beck Depression Inventory-II; SAS, Self-rating Anxiety Scale; HAMD, Hamilton Depression Rating Scale; HAMA, Hamilton Anxiety Rating Scale.

### Plasma lipid profiles and plasma levels of FGF19 and FGF21 in the clinical study

**Figure 1:**
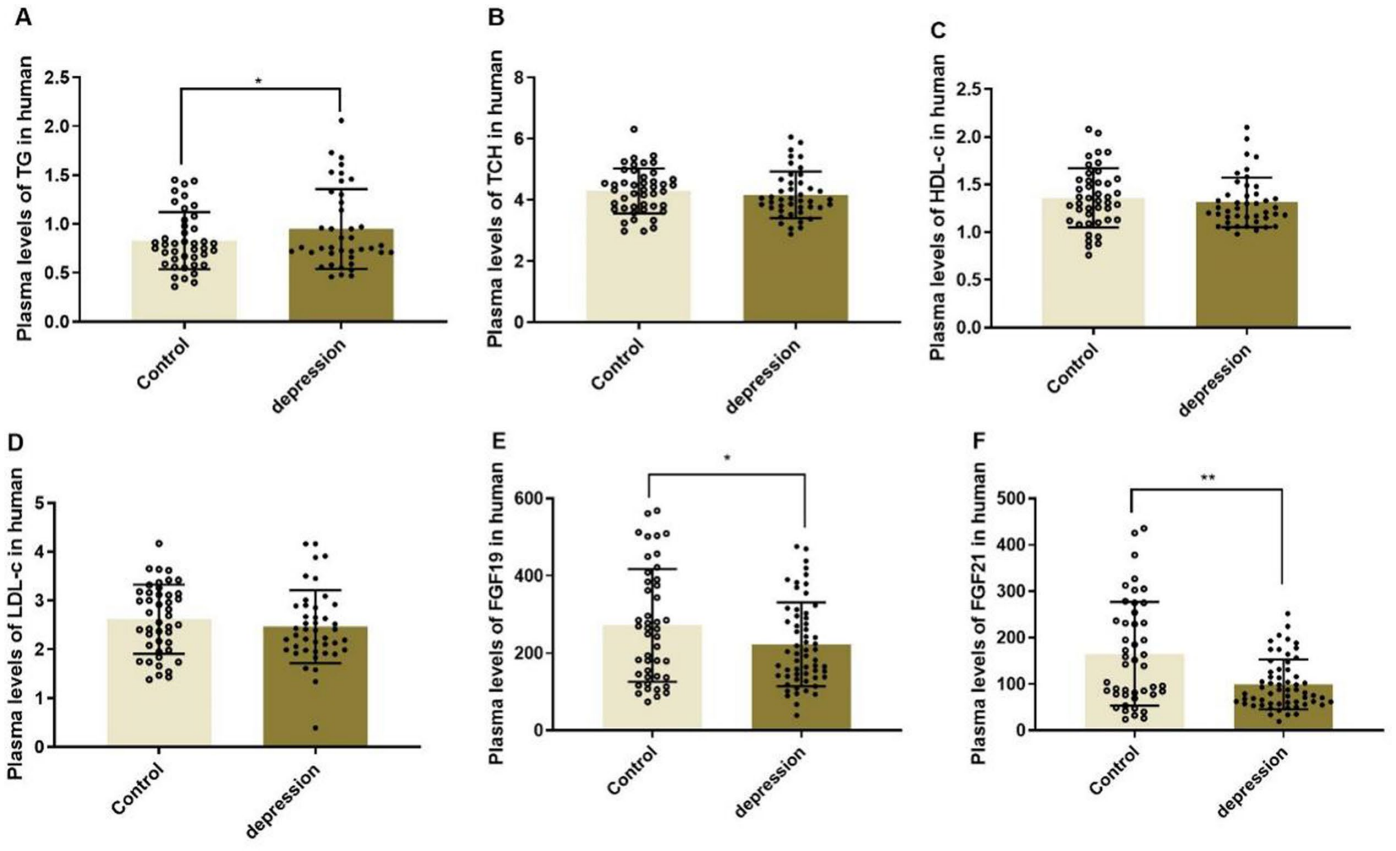
Plasma levels of lipid profiles (A-D) and FGF19 (E) and FGF21 (F) in MDD patients and healthy controls, ^*^p < 0.05, ^**^p < 0.01. TG, triglyceride; TCH, total cholesterol; HDL-c, high-density lipoprotein-cholesterol; LDL-c, low-density lipoprotein-cholesterol.

The MDD patients presented significantly lower plasma levels of FGF19 and FGF21 than healthy controls as shown in Figure 1 (271.89 ± 146.04 vs. 222.69 ± 108.34, *p* < 0.05 and 165.17 ± 111.81 vs. 99.84 ± 53.69, *p* < 0.01). Meanwhile, the plasma lipid profiles, including TCH, HDL-c, and LDL-c, had no significant differences between MDD patients and controls, except for TG, which was significantly elevated in MDD patients (*p* < 0.05).

### Gene expression of PGC-1α and FNDC5 in the clinical study

**Figure 2:**
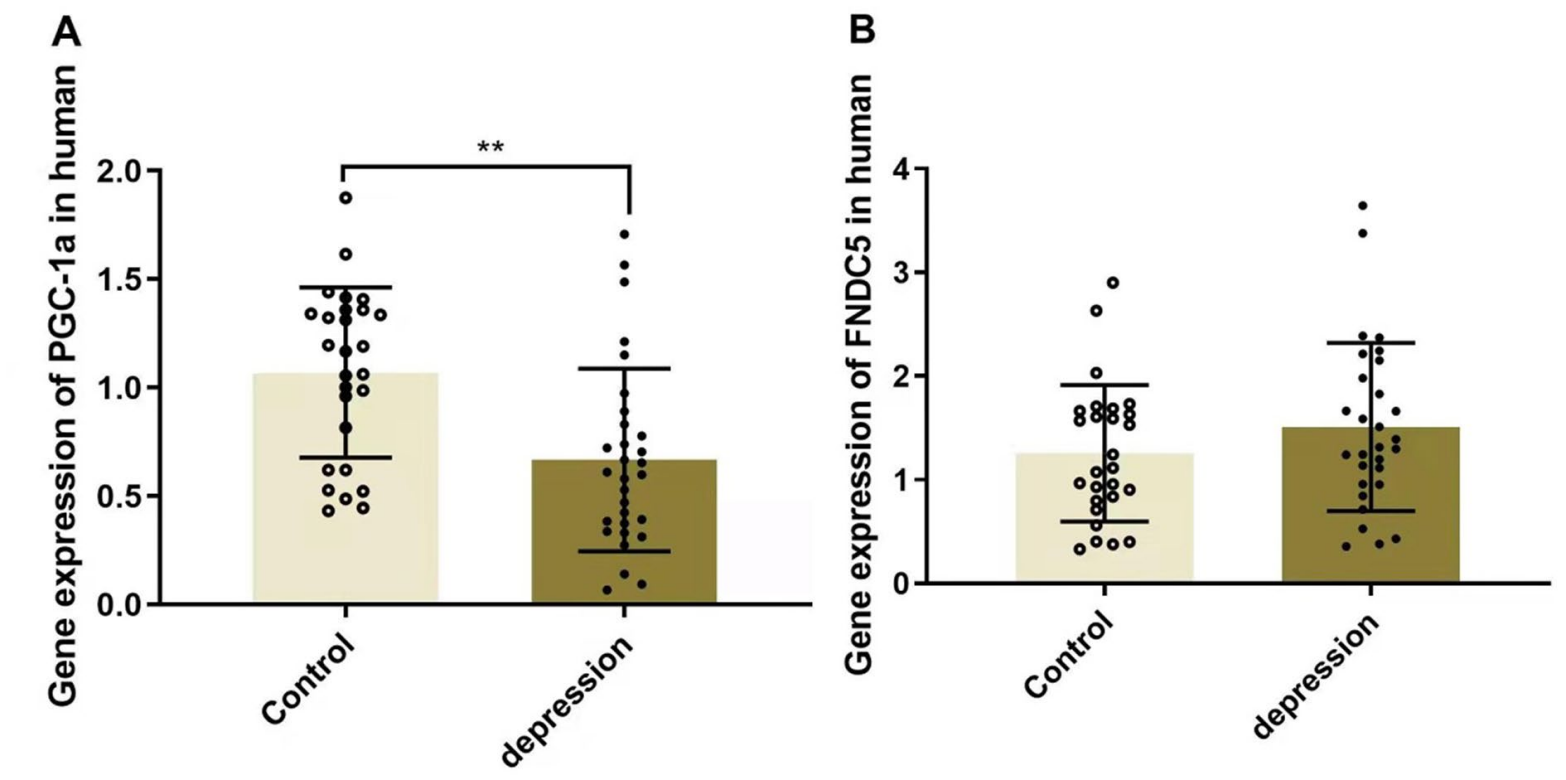
Gene expressions of PGC-1α (A) and FNDC5 (B) in MDD patients and healthy controls, ^**^p < 0.01. PGC-1α, peroxisome proliferator-activated receptor γ coactivator α; FNDC5, fibronectin type III domain—containing protein 5.

As shown in Figure 2, the serum level of PGC-1α was significantly downregulated in MDD patients (1.070 ± 0.151 in HCs and 0.667 ± 0.253 in depression, *p* < 0.01), whereas FNDC5 levels remained stable (1.256 ± 0.226 in HCs and 1.509 ± 0.442 in depression, *p* > 0.05).

### Relationship between plasma levels of FGF19 and FGF21 and plasma lipid profiles

As shown in Table 3, Pearson correlation analyses showed that TG was negatively correlated with FGF19 levels (*r* = −0.3958, *p* = 0.0205) in patients with depression, while no significant relationship was found between FGF21 levels and lipid profiles.

**Table 3:** Correlation analysis between FGF19, FGF21, and lipid profiles.

	TCH	TG	HDL-C	LDL-C
Plasma levels of FGF19 in total	0.04439 (0.6903)	0.06629 (0.5516)	−0.04760 (0.6691)	0.06630 (0.5515)
Plasma levels of FGF21 in total	−0.0807 (0.4911)	0.1421 (0.2239)	−0.1423 (0.2234)	−0.0049 (0.9667)
Plasma levels of FGF19 in HCs	0.04024 (0.7953)	0.1664 (0.2803)	−0.1649 (0.2849)	0.1205 (0.4360)
Plasma levels of FGF21 in HCs	−0.2082 (0.1750)	0.2119 (0.1673)	−0.2255 (0.1412)	−0.1152 (0.4567)
Plasma levels of FGF19 in patients with depression	0.0173 (0.9167)	**−0.3958 (0.0205)**	0.1508 (0.3595)	−0.2800 (0.1034)
Plasma levels of FGF21 in patients with depression	0.1017 (0.5862)	0.0573 (0.7596)	−0.0196 (0.9167)	0.1221 (0.5128)

TCH, total cholesterol; TG, triglyceride; HDL-c, high-density lipoprotein-cholesterol; LDL-c, low-density lipoprotein-cholesterol; HCs, healthy controls.

### Relationship between plasma levels of FGF19 and FGF21 and clinical symptom scores

The associations between FGF19 and FGF21 levels and clinical symptom scores of the total population, HCs, and patients with depression were analyzed separately. In the total population, the Pearson correlation showed no association between FGF19 and FGF21 levels and clinical symptom scores. In HCs, we found a statistically significant positive association between FGF21 levels and BDI-II (*r* = 0.5184, *p* = 0.0014) and SAS (*r* = 0.1661, *p* = 0.0288) scores. Moreover, in patients with depression, a positive association was found between FGF19 levels and all clinical symptom scores, i.e., BDI-II, SAS, HAMD, and HAMA scores (Table 4).

**Table 4:** Correlation analysis between FGF19 and FGF21 levels and clinical symptom scores.

	BDI-II	SAS	HAMD	HAMA
Plasma levels of FGF19 in total	−0.0528 (0.6443)	0.0066 (0.9518)	–	–
Plasma levels of FGF21 in total	−0.0427 (0.7273)	−0.0812 (0.4795)	–	–
Plasma levels of FGF19 in HCs	−0.0167 (0.9241)	0.0283 (0.8588)	–	–
Plasma levels of FGF21 in HCs	**0.5184 (0.0014)**	**0.1661 (0.0288)**	–	–
Plasma levels of FGF19 in patients with depression	**0.3637 (0.0194)**	**0.3862 (0.0096)**	**0.3898 (0.0107)**	**0.4344 (0.0040)**
Plasma levels of FGF21 in patients with depression	−0.0285 (0.8727)	−0.0222 (0.8969)	0.0465 (0.7879)	0.1535 (0.3714)

BDI-II, Beck Depression Inventory-II; SAS, Self-rating Anxiety Scale; HAMD, Hamilton Depression Rating Scale; HAMA, Hamilton Anxiety Rating Scale; HCs, healthy controls.

### Relationship between plasma levels of FGF19 and FGF21 and cognitive performance

Pearson correlation analyses revealed no correlation between levels of FGF19 and FGF21 and participants’ gender, age, education years, or BMI in both the MDD group and HC group. Our previously published article revealed that immediate memory, delayed memory, and RBANS total score were significantly decreased in depressed patients compared with healthy controls (Wang et al., 2020). Furthermore, the correlation analysis in this article showed that serum FGF19 and FGF21 levels were correlated with immediate memory (*r* = 0.2592, *p* = 0.0268 and *r* = 0.2410, *p* = 0.0254, respectively). FGF19 was negatively correlated with language (*r* = −0.2384, *p* = 0.0408). FGF21 was negatively correlated with attention (*r* = −0.2302, *p* = 0.0351) and delayed memory (*r* = −0.3008, *p* = 0.0057) in RBANS indexes. However, there was no correlation between FGF19 and FGF21 levels and the RBANS scale in HCs (Table 5).

**Table 5:** Correlation analysis between FGF19 and FGF21 levels and RBANS in depression.

	Correlation with RBANS score (r)				
	Immediate memory	Visuospatial /constructional	Language	Attention	Delayed memory
	Index	Index	Index	Index	Index
FGF19	**0.2592 (0.0268)**	0.0948 (0.4091)	**−0.2384 (0.0408)**	0.0172 (0.8810)	−0.0939 (0.4137)
FGF21	**0.2410 (0.0254)**	−0.06773 (0.5331)	0.02932 (0.7912)	**−0.2302 (0.0351)**	**−0.3008 (0.0057)**

RBANS, repeatable battery for the assessment of neuropsychological status.

### Relationship between plasma levels of FGF19 and FGF21 and gene expression of PGC-1α and FNDC5

Correlation analyses showed a significant negative association between plasma levels of FGF19 and PGC-1α (*r* = −0.301, *p* < 0.05) in MDD patients, while no significant association was found between FGF19 and FNDC5 (Table 6). As for FGF21, it was positively associated with PGC-1α and negatively associated with FNDC5.

**Table 6:** Correlation analysis between FGF 19 and PGC-1α/FNDC5 in depression.

	PGC-1α	FNDC5
FGF19	**−0.301 (0.0376)**	0.0205 (0.8899)
FGF21	**0.3743 (0.0095)**	**−0.2947 (0.0378)**

PGC-1α, peroxisome proliferator-activated receptor γ coactivator α; FNDC5, fibronectin type III domain – containing protein 5.

## Discussion

Our study first demonstrated that plasma FGF19 and FGF21 levels were significantly decreased in patients with MDD compared to healthy controls. Then, we analyzed their relationship with metabolic and cognitive dysregulation in depression. We found a significant negative association between TG and FGF19 levels. Moreover, both FGF19 and FGF21 levels were positively correlated with immediate memory. However, FGF19 levels were negatively correlated with language, and FGF21 levels were also negatively correlated with attention and delayed memory in RBANS indexes. Finally, we further explored the underlying mechanisms and elucidated that the PGC-1α/FNDC5 pathway might be responsible for depression with co-morbid cognitive impairment.

We elucidated that expression levels of FGF19 were decreased in patients with MDD, suggesting that they might serve as a potential peripheral biomarker for MDD. In a previous study on nicotine dependence male subjects (Liu et al., 2017b), the FGF19 levels in CSF were found positively related to the score of BDI. Precisely, FGF19 levels were shown to affect sleep and negative emotion-related behaviors in subjects. Similarly, our study also discovered a positive association between FGF19 levels and BDI in patients with MDD. Moreover, we further investigated the association between FGF19 and other scales, such as SAS, HAMD, and HAMA, and came to the similar conclusion that the plasma FGF19 levels were also positively correlated with other scales’ scores, which provide evidence of the role of FGF19 in mood regulation including both depressive and anxiety behaviors in patients with depression. FGF21, another endocrine FGF and a key mediator of the effects of mood-stabilizing agents (Leng et al., 2015), was also observed to decrease significantly in patients with MDD. FGF21 in CSF was previously reported to have a negative correlation with BDI in male subjects rather than female subjects (Liu et al., 2017a). Previous studies have shown different roles of FGF21 between genders due to different estrogen levels (Zhang et al., 2015), and FGF21 was negatively correlated with estrogen in diabetic women. Our finding that FGF21 levels have no correlation with BDI scores in the total population may be explained by this gender difference. Particularly worth mentioning is that FGF21 has been demonstrated to cross the blood–brain barrier in rats (Sarruf et al., 2010; Hsuchou et al., 2013), and there is a linear relationship between serum and CSF levels. Thus, we chose to investigate the plasma levels in the present study which were available in the clinical study.

Previous clinical studies indicated that MDD patients are prone to lipid metabolic dysregulation (Sagud et al., 2009; Hummel et al., 2011; Scharnholz et al., 2014). Our clinical study showed that the plasma TG of MDD patients increased significantly compared with healthy controls, reflecting dyslipidemia in MDD. The correlation analysis showed a significant negative association between TG and FGF19 levels. Because FGF19 suppresses the synthesis of TG (Degirolamo et al., 2016), an increased TG level in MDD patients may be related to a decreased FGF19 level. FGF21 induces a broad range of favorable metabolic effects. The pharmacological administration of FGF21 exerted metabolic modulating effects, including enhancing insulin sensitivity, decreasing plasma TG and LDL-c concentrations, and increasing HDL-c (Kharitonenkov and Adams, 2013). In this study, however, we found no significant relationship between FGF21 levels and lipid profiles. Bipolar disorder (BD) is another mental disorder that is also characterized by metabolic and cognitive dysfunctions. In a study of BD (Chang et al., 2018), FGF21 level and metabolic indices did not differ significantly between the controls and patients, but after 12 weeks of treatment, the FGF21 level had increased significantly and the change in FGF21 level was correlated with the changes in HAMD, TCH, and LDL-c. Therefore, we consider that the relationship between FGF21 levels and lipid profiles may change after antidepressant treatment. In the following study, we plan to observe changes in FGF19/FGF21 levels after treatment using antidepressants in MDD patients to further validate the role of FGF19/FGF21 in MDD patients.

In our previous article (Wang et al., 2020), we conducted an observational case–control study to compare cognitive function between depressed patients and healthy controls and found that immediate memory, delayed memory, and RBANS total score were significantly decreased in depressed patients. In this continuous study, correlation analyses indicated that both FGF19 and FGF21 levels were positively correlated with immediate memory. However, FGF19 levels were negatively correlated with language and FGF21 levels were also negatively correlated with attention and delayed memory in RBANS indexes. Omileke et al. (2020) found significant positive correlations between plasma FGF21 levels and some cognitive parameters, such as word association in patients with BD, indicating that higher peripheral FGF21 might be associated with better cognitive performance. Moreover, FGF21 administration was proven to improve cognitive dysfunction and anxiety-like behavior in obese rats (Sa-nguanmoo et al., 2018; Wang et al., 2018). Unlike FGF21, there are no prior research studies assessing the relationship between FGF19 levels and cognitive performance. Our results revealed that decreasing FGF19 and FGF21 levels were associated with cognitive impairment in MDD patients, which need further verification.

We further explored the possible mechanisms by which FGF19 and FGF21 affect metabolism and cognition. Correlation analysis showed a significant negative association between plasma levels of FGF19 and PGC-1α. As for FGF21, it is positively associated with PGC-1α and negatively associated with FNDC5. As we mentioned above, it is known that FGF19 and FGF21 can act through the PGC-1α/FNDC5 pathway. FGF19 can act against obesity-induced muscle atrophy, metabolic derangement, and abnormal irisin secretion by promoting PGC-1α and FNDC5 expression (Guo et al., 2021). In addition, FGF21-associated upregulation of PGC-1α expression was reported to play an important role in metabolic remodeling (Kobayashi et al., 2020), but no studies have evaluated the influence of this pathway on cognitive impairment so far. Previous studies mentioned earlier have linked the alteration of the PGC-1α/FNDC5 pathway with BDNF expression in the brain. BNDF promotes many aspects of brain development and neuroplasticity that underlie cognitive function (Kuipers and Bramham, 2006; Greenberg et al., 2009). A clinical study proved that individuals with BDNF val66met genotype exhibit decreased secretion of BDNF, accompanied by deficits in episodic memory function and increased risk of anxiety and depression (Egan et al., 2003; Hariri et al., 2003). In patients with post-stroke depression, their serum BNDF level is lower than in those without depression, and antidepressants could enhance the BDNF expression in their brains (Zhang and Liao, 2020). Thus, we speculate that FGF19 and FGF21 may play a role in cognitive dysregulation in MDD by modulating the BDNF expression.

In summary, this study elucidated the role of FGF19 and FGF21 in MDD. Moreover, metabolic and cognitive dysregulation in MDD patients had been evaluated and linked to the decreased concentrations of FGF19 and FGF21 through the PGC-1α/FNDC5 pathway. Our results showed the alterations of FGF19 and FGF21 levels may be a common pathogenic mechanism of the metabolic and cognitive disturbances in patients with MDD and identifying these mechanisms could potentially represent novel therapeutic targets or individual-specific strategies to combat MDD.

## Data availability statement

The original contributions presented in the study are included in the article/supplementary material, further inquiries can be directed to the corresponding author.

## Ethics statement

The studies involving human participants were reviewed and approved by clinicaltrials-NCT03295708. The patients/participants provided their written informed consent to participate in this study.

## Author contributions

RD designed and performed the experiments and amend the manuscript. MT and SC analyzed, interpreted the data, and wrote the manuscript. LW and HT performed the experiments, collected, and interpreted the data. TL and TZ undertook the statistical analysis. All authors have read and approved the manuscript.

## Funding

This study was supported by the National Natural Science Foundation of China (No. 81803233 and 81703625), the fellowship of China Postdoctoral Science Foundation (2021M693561), the Natural Science Foundation of Hunan Province (No. 2018JJ3834), and the Science Foundation of Xiangya Hospital for Young Scholar (No. 2017Q13).

## Conflict of interest

The authors declare that the research was conducted in the absence of any commercial or financial relationships that could be construed as a potential conflict of interest.

## Publisher’s note

All claims expressed in this article are solely those of the authors and do not necessarily represent those of their affiliated organizations, or those of the publisher, the editors and the reviewers. Any product that may be evaluated in this article, or claim that may be made by its manufacturer, is not guaranteed or endorsed by the publisher.
